# Potential of Eucalyptus Oil as Repellent against House Rat, *Rattus rattus*

**DOI:** 10.1155/2014/249284

**Published:** 2014-01-12

**Authors:** Neena Singla, Ramandeep Kaur Thind, Amrit Kaur Mahal

**Affiliations:** ^1^Department of Zoology, Punjab Agricultural University, Ludhiana 141004, India; ^2^Department of Mathematics, Statistics and Physics, Punjab Agricultural University, Ludhiana 141004, India

## Abstract

Rodent repellents are chemicals which by taste or odour or possibly by both will prevent animal from feeding or gnawing. Such substances may be used in protecting an area from rodent infestation or in protecting packaged food, packing materials, electric cables, and other important vulnerable materials. Mature and healthy house rat, *Rattus rattus* of both sexes, was exposed to 5, 10, and 20% eucalyptus oil applied as spray in laboratory pens in bichoice tests. Each concentration was applied through three different modes of application, that is, daily, once, and alternatively in a week. Repellent effect of the oil was assessed based on food consumption from treated and untreated sides for four days. In overall, food consumption was significantly (*P* < 0.0001) low from treatment side compared to the untreated side indicating significant repellent effect of the oil at all the three concentrations tested. Repellent effect of the oil was, however, not found to differ significantly between the two sexes. Percent repellency in both male and female rats was apparently more with daily application of 5 and 10% eucalyptus oil. Present studies reveal the potential of eucalyptus oil in repelling away *R. rattus*; however, further studies may be conducted to enhance the persistence of repellent effect for longer period of time.

## 1. Introduction

Rodents have gained the reputation as one of the most persistent and ubiquitous vertebrate pests affecting human populations. They cause economic problems because of the damage they inflict on agricultural systems [1, 2], environmental problems due to the chemicals used for their control [3], social problems associated with their close proximity to human habitation [4], and health problems as carriers of zoonoses [5–7]. The house rat, *Rattus rattus* Linnaeus, 1758 (Rodentia: Muridae), is one of the most commonly encountered and economically important commensal rodents. It not only inflicts heavy damage to stored food but also have nuisance value being a disease carrier or vector. It is purely an indoor pest [8, 9]. Conventional pesticides possess inherent toxicities that endanger the health of the farm operators, consumers, and the environment. Such negative effects of conventional pesticides on human health led to a resurgence in interest in botanical pesticides because of their minimal costs and fewer ecological side effects. Botanicals have advantages over broad-spectrum conventional pesticides. They affect only target pest and closely related organisms, are effective in very small quantities, decompose quickly, and provide the residue-free food and a safe environment to live. Natural products represent one of the most important alternatives to control pests and diseases that affect plants and animals without deleteriously affecting environmental safety [10–12].

Plants with strong smells act as repellents and can protect the crops nearby [13–15]. Singla and Parshad [16] studied the antifeeding effects of neem-based formulation against *R. rattus*. Parshad et al. [17] reported repellent effect of two fungicides against *R. rattus. *Kalandakanond-Thongsong et al. [18] evaluated the efficacy of chilli, wintergreen oil, bergamot oil, peppermint oil, and geranium oil as repellents in the circular open field against adult male Wistar rats. Pine needle oil inhibits feeding in vertebrate species through sensory cues [19]. Some botanicals also have antireproductive effects against pests [20–22] while some have positive effects on growth [23].

Among the plant families with promising essential oils used as repellents include *Cymbopogon* spp., *Ocimum* spp., *Thymus* spp., and *Eucalyptus* spp. [24]. Among essential oils, eucalyptus oil, in particular, is more useful as it is easily extractable commercially (industrial value) and possesses a wide range of desirable properties worth exploiting for pest management [25–27]. The oil is a colourless liquid, with a camphor-like odour and spicy, cooling taste. It possesses a wide spectrum of biological activity including antimicrobial, fungicidal, insecticidal/insect repellent, herbicidal, acaricidal, and nematicidal [28]. The pesticidal activity of eucalyptus oil has been due to the components such as 1, 8-cineole, p-cymene, eucamalol, limonene, linalool, *α*-pinene, *γ*-terpinene, *α*-terpineol, alloocimene, and aromadendrene [29, 30]. The use of eucalyptus oil as a natural pesticide is of immense significance in view of the environmental and toxicological implications of the indiscriminate use of synthetic pesticides and overcoming/reducing the problem of increasing pest resistance [28].

Rodent repellents are chemicals which by taste or odour or possibly by both will prevent animal from feeding or gnawing. Such substances may be used in protecting an area from rodent infestation or in protecting packaged food, packing materials, electric cables, and other important vulnerable materials. Relatively little work has been carried out on plant-derived repellents compared to other aspects of rodent control. No study has yet been made on evaluating the potential of eucalyptus oil as repellent against rodent pests. The present study was hence carried out to evaluate the potential of eucalyptus oil as repellent against *R. rattus*, a predominant rodent pest species.

## 2. Material and Methods

The present work was carried out in Animal House Laboratory, Department of Zoology, Punjab Agricultural University, Ludhiana, India, located at an intersection of 30°55′N parallel of latitude and 75°54′E line of longitude. Commercially available pure eucalyptus oil was used for the present study.

### 2.1. Collection and Maintenance of Animals

For present studies, *R. rattus* of both sexes were trapped with the help of single catch and multicatch rat traps from store houses and poultry farms in and around Ludhiana. In the laboratory, rats were acclimatized individually in cages of size 36×23×23 cm each for 15–20 days before the commencement of experiment. Food and water were provided *ad libitum*. Food consisted of a mixture of cracked wheat, powdered sugar and groundnut oil (WSO) in ratio 96 : 2 : 2. The metallic trays were kept under each cage for the collection and disposal of urine and faeces. Animals were used and maintained as per the guidelines of Institutional Animal Ethics Committee. After acclimatization, healthy and mature rats of both sexes were weighed and selected for experimentation.

### 2.2. Experimental Setup

A total of four laboratory pens (each of size 252×100×72 cm) were used for each experiment. Each laboratory pen consisted of three chambers of equal size. One rat was released in each chamber. Each chamber in a laboratory pen, on its opposite facing sides, was connected to two small nest boxes (each of size 20×15×15 cm) by means of holes (each of diameter 6 cm). Each rat had free access to the two nest boxes attached on opposite sides of a chamber. Treatment was carried out in the nest box of one side of a chamber. Oil was sprayed on all the inner sides of the nest box.

### 2.3. Treatment

Three different concentrations of eucalyptus oil, that is, 5, 10, and 20%, were tested. Different concentrations of the oil were prepared by in isopropyl alcohol. Each concentration was tested on a total of twelve rats (six of each sex) by applying as spray (using a small spray pump of 100 mL capacity). Rats were exposed to each concentration of the oil through three different modes of application, that is, applied daily (from Monday to Thursday), applied once a week (on Monday only), and applied alternatively (on Monday, Tuesday, and Thursday). Weighed (20 g) amount of food, that is, WSO taken in a bowl was placed in both the nest boxes of each chamber.

### 2.4. Repellent Effect

Repellent effect of the oil was assessed based on the consumption of food by the rat from the food bowls kept in two nest boxes of a chamber in a laboratory pen. Food consumption was recorded daily after every 24 h from both treated and untreated sides for 4 days in a week, that is, from Tuesday to Friday to determine mean daily food consumption (g/100 g body weight (bw)). Based on mean daily food consumption data, percent repellency was determined using the formula given below

(1) $$\begin{array}{c} \text{percent}\;\text{repellency}=\frac{\text{FUT}-\text{FT}}{\text{FUT}}\times 100, \end{array}$$

where FUT is the mean daily food consumption from untreated side and FT is the mean daily food consumption from treated side.

### 2.5. Statistical Analyses

Values were determined as mean ± SD. The data on food consumption for two sexes, three concentrations of the oil, three modes of applications, four days of application, and from treatment and untreated sides was collected using factorial experiments in completely randomized design. Analysis was done using general linear model (GLM) in SAS 9.3. All pairwise treatment comparisons were made using Tukeys' HSD test at 5% level of significance.

## 3. Results

**Figure 1:**
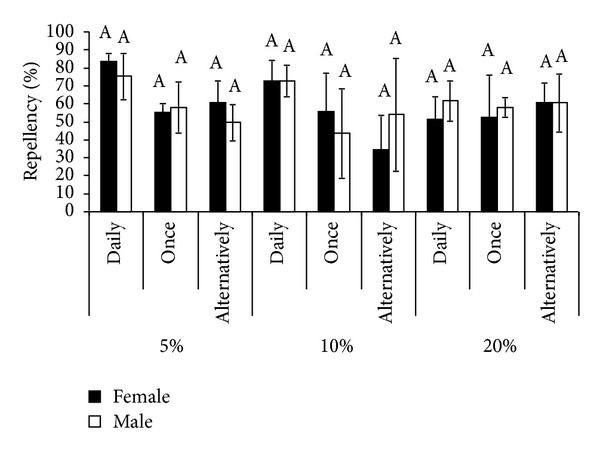
No significant difference in average percent repellency of eucalyptus oil in *Rattus rattus* between male and female rats at three different concentrations with three different modes of application each. Bars with similar superscripts differ nonsignificantly.

Statistical analysis of the data revealed in overall significantly (*P* < 0.0001) low consumption of food from treatment side compared to untreated side at all three concentrations and modes of application (Tables 1–3) indicating repellency of eucalyptus oil when applied as spray. Significant difference in food consumption from treatment side was observed among all three concentrations tested (*P* = 0.0186). No significant difference in average percent repellency of all the three concentrations of the oil was observed between male and female rats at all the three modes of application (Figure 1).

### 3.1. Effect of 5% Eucalyptus Oil

The average mean daily consumption of food by female rats was significantly (*P* ≤ 0.05) low from treatment side when 5% oil was applied daily and alternatively (Table 1); however, when the oil was applied once a week, the average mean daily food consumption by female rats was found to be nonsignificantly low from treatment indicating low repellency of the oil at this mode of application. This may be due to the dissipation of repellent effect of the oil which was applied only on day 1 of the week. The average mean daily food consumption in male rats was significantly low from treatment side at all the three modes of application (Table 1).

**Figure 2:**
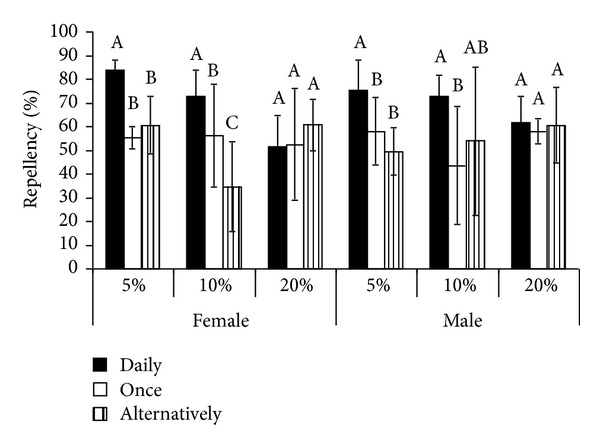
Significant differences in average percent repellency of eucalyptus oil in *Rattus rattus* among three different modes of application at three different concentrations in both male and female rats. Bars with differ superscripts different significantly at *P* ≤ 0.05.

Average percent repellency with 5% eucalyptus oil applied as spray, was found to be significantly (*P* ≤ 0.05) high when the oil was applied daily (mode I) in both male and female rats (Table 4). The difference in average percent repellency between the modes II and III was not found to differ significantly in both the sexes (Figure 2).

### 3.2. Effect of 10% Eucalyptus Oil

When 10% eucalyptus oil was applied as spray, the average mean daily consumption of food was found to be significantly (*P* ≤ 0.05) low from treatment side when the oil was applied daily in male rats, whereas, in female rats the consumption was found to be significantly low when applied daily as well as when applied once a week (Table 2). The average consumption of four days was nonsignificantly low in females when the oil was applied alternatively and in males when the oil was applied once a week and alternatively. In both male and female rats, the average consumption of treatment food was not found to differ significantly among the three modes of application.

In female rats, significant difference in average percent repellency with 10% eucalyptus oil was found among the three modes of application (Figure 2). It was high when the oil was applied daily followed by when applied once a week and alternatively (Table 4). In male rats, a significant difference in average percent repellency was found between modes I and II, however, similar differences between modes I and III and between modes II and III were found to be non-significant (Figure 2). Percent repellency was high when the oil was applied daily followed by when applied alternatively and once a week (Table 4). No significant difference in percent repellency was observed among the four days of treatment at all three modes of application in female rats.

However, in male rats, percent repellency on day 3 of treatment was found to be significantly (*P* ≤ 0.05) low from that observed on day 1 of treatment when the oil was applied alternatively. The same was significantly low on days 3 and 4 from that observed on day 1 when the oil was applied once a week (Table 4). This may again be due to dissipation of repellent effect of the oil applied on day 2 in mode III and on day 1 in mode II of application, respectively.

### 3.3. Effect of 20% Eucalyptus Oil

The average mean daily food consumption was found to be significantly (*P* ≤ 0.05) low from treatment side compared to untreated side in female rats when 20% eucalyptus oil was applied daily and once a week (Table 3). Similar difference in female rats was found to be non-significant when the oil was applied alternatively. In male rats, the average mean daily food consumption was found to be significantly (*P* ≤ 0.05) low from treatment side compared to untreated side when the oil was applied daily. The similar differences in male rats were non-significant when the oil was applied once a week and alternatively (Table 3). The average mean daily food consumption of treatment food among the three modes of application was found to differ nonsignificantly in both the sexes.

The average percent repellency was not found to differ significantly among the three modes of application in both male and female rats (Figure 2). In female rats, when 20% eucalyptus oil was applied daily and alternatively, difference in percent repellency among the four days of treatment was not found to be significant, however, when the oil was applied once a week, percent repellency was significantly less on day 3 compared to day 1 and also on day 4 compared to days 1 and 2 (Table 4).

In male rats, no significant difference in percent repellency was observed among four days of application at all three modes of application. No significant difference in percent repellency was observed between male and female rats when oil was applied daily and alternatively. Significant (*P* ≤ 0.05) difference was; however, observed on day 4 when oil was applied once a week with higher repellency in case of male rats (49.67%) from that observed in female rats (20.50%). The average percent repellency at all the modes of application was not found to differ significantly between the rats of two sexes (Table 4).

## 4. Discussion

During the present studies, significant differences were found in mean daily food consumption from treatment and untreated sides at different modes of application and at different concentrations of the oil between male and female rats. This may be due to the sex specific variation in response to *R. rattus* towards the oil. Similar sex specific differences in response to *R. rattus* of two sexes towards toxic baits were reported by Kaur et al. [31]. During the present studies, higher standard deviation values than mean values of food consumption were observed in some cases which may be due to individual variation in response shown by rats of different species [32].

In overall, no significant difference in repellent effects of oil applied as spray was found between the two sexes and among the three concentrations of the oil tested during the present studies. All the three concentrations were equally effective and the repellency was highest when the oil was applied daily. The cost of spraying 5% eucalyptus oil (the minimum effective concentration) per 1 m^2^ area comes out to be Indian Rs 10 (US $ 0.17), which can be considered cost effective if we keep in view the extent of loss caused by *R. rattus* through damage and contamination of food. Among the various components of eucalyptus oil, 1, 8-cineole is the most important one largely responsible for a variety of its pesticidal properties [29]. The presence of this essential oil also provides defense advantage to eucalyptus leaves against herbivory and attack by harmful insects [33]. The present study is the first of its kind evaluating repellent potential of eucalyptus oil against vertebrate pests. Previous to this, repellency of eucalyptus oil has been recorded against the tick, *Ixodes ricinus* [34] and against acaricide-resistant mites [35]. Application of 1.0% concentration of 1, 8 cineole reduced oviposition rate of *Thrips tabaci* by 30–50% as compared to untreated controls [36]. Eucalyptus oil (1%) added to sugar syrup, repelled honey bees [37]. Eucalyptus oil (2%) on filter paper and wood floor repelled termites [38]. Eucalyptus oil can also protect plants against rice weevils, pine processionary moths, and mushroom flies [28]. Essential oils of eucalyptus appear particularly potent as mosquito repellents [39].

Since eucalyptus oil possesses a wide spectrum of biological activity and is regarded as safer compounds, there have been attempts to commercialize and market the insecticides/repellent products containing eucalyptus oil as such or based upon them. Quwenling is a eucalyptus-based product that has been successfully marketed as an insect-repellent in China [40]. It provides protection against *Anopheles* mosquitoes parallel to DEET and has, in fact, replaced the widely used synthetic repellent, dimethyl phthalate.

## 5. Conclusion

The present studies reveal the potential of eucalyptus oil applied as spray in repelling away *R. rattus* of both sexes. Percent repellency was more when the oil was applied daily and alternatively as compared to when applied once a week indicating low persistence of the repellent effect due to volatile nature of the oil. Further studies may be conducted to prepare formulations of the oil leading to slow release of the oil so as to increase its persistence for a longer period of time.

## Figures and Tables

**Table 1:** Mean daily food consumption by *Rattus rattus *in response to 5% eucalyptus oil applied as spray.

Mode of application	Days of application	Mean daily food consumption (g/100 g bw)			
		Female rats (*n* = 6) (body wt = 121.66 ± 17.71 g)		Male rats (*n* = 6) (body wt = 155.00 ± 22.91 g)	
		Treatment side	Untreated side	Treatment side	Untreated side
I	Day 1	2.00 ± 3.61^a^	12.77 ± 1.40^b^	2.04 ± 2.94^a^	10.62 ± 3.45^b^
	Day 2	1.41 ± 1.09^a^	14.03 ± 3.14^b^	2.24 ± 2.03^a^	10.18 ± 2.73^b^
	Day 3	2.00 ± 2.98^a^	10.27 ± 5.77^b^	3.04 ± 3.72^a^	7.01 ± 3.03^b^
	Day 4	1.28 ± 1.93^a^	11.36 ± 2.97^b^	1.18 ± 2.06^a^	9.17 ± 1.99^b^
	Average	1.67 ± 0.33^**A**^	12.10 ± 1.42^**B**^	2.12 ± 0.66^**A**^	9.24 ± 1.39^**B**^

II	Day 1	3.51 ± 2.73^a^	9.98 ± 5.98^b^	1.82 ± 2.00^a^	9.25 ± 2.83^b^
	Day 2	8.18 ± 6.16^a^	5.31 ± 4.70^b^	3.38 ± 2.74^a^	7.82 ± 2.48^b^
	Day 3	5.51 ± 6.71^a^	7.55 ± 5.14^b^	2.90 ± 3.02^a^	9.46 ± 2.91^b^
	Day 4	5.75 ± 4.50^a^	7.74 ± 4.54^b^	5.76 ± 3.88^a^	8.05 ± 3.40^b^
	Average	5.73 ± 1.65^**BC**^	7.64 ± 1.65^**C**^	3.46 ± 1.44^**A**^	8.64 ± 0.71^**B**^

III	Day 1	5.27 ± 1.35^a^	10.71 ± 5.05^b^	4.75 ± 2.05^a^	7.82 ± 3.23^b^
	Day 2	2.21 ± 2.43^a^	8.44 ± 5.57^b^	3.40 ± 1.89^a^	9.37 ± 2.39^b^
	Day 3	3.88 ± 3.58^a^	8.68 ± 5.17^b^	4.80 ± 4.01^a^	4.68 ± 3.31^b^
	Day 4	2.75 ± 2.89^a^	9.36 ± 4.39^b^	2.71 ± 1.55^a^	7.76 ± 2.46^b^
	Average	3.52 ± 1.17^**A**^	9.29 ± 0.88^**C**^	3.91 ± 0.89^**A**^	7.40 ± 1.70^**B**^

Values are mean ± SD, I = daily, II = once a week, and III = alternatively.

Values with similar superscripts in a column for four days of application (a or b) and for average values (A, B, or C) at each mode of application indicate no significant difference.

Values with different superscripts in a row for each sex for four days of application (a-b) and for average values (A–C) at each mode of application indicate significant difference at *P* ≤ 0.05.

**Table 2:** Mean daily food consumption by *Rattus rattus *in response to 10% eucalyptus oil applied as spray.

Mode of application	Days of application	Mean daily food consumption (g/100 g bw)			
		Female rats (*n* = 6) (body wt = 156.66 ± 24.94 g)		Male rats (*n* = 6) (body wt = 143.33 ± 22.85 g)	
		Treatment side	Untreated side	Treatment side	Untreated side
I	Day 1	3.64 ± 4.25^a^	10.49 ± 3.99^b^	1.40 ± 2.83^a^	12.74 ± 2.93^b^
	Day 2	0.80 ± 0.91^a^	11.45 ± 1.97^b^	2.11 ± 2.74^a^	9.50 ± 3.10^b^
	Day 3	4.90 ± 5.32^a^	13.37 ± 2.76^b^	5.26 ± 5.18^a^	10.72 ± 2.99^b^
	Day 4	4.10 ± 4.49^a^	14.18 ± 3.94^b^	3.85 ± 5.56^a^	11.67 ± 2.60^b^
	Average	3.36 ± 1.54^**A**^	12.37 ± 1.47^**B**^	3.15 ± 1.50^**A**^	11.15 ± 1.19^**B**^

II	Day 1	3.47 ± 5.56^a^	14.23 ± 4.03^b^	2.42 ± 3.82^a^	12.07 ± 5.33^b^
	Day 2	2.60 ± 4.22^a^	12.67 ± 6.12^b^	4.96 ± 5.18^a^	11.57 ± 5.78^b^
	Day 3	4.85 ± 2.89^a^	8.99 ± 6.10^b^	9.76 ± 6.13^a^	3.40 ± 4.28^b^
	Day 4	9.35 ± 7.46^a^	8.85 ± 2.50^b^	7.31 ± 4.70^a^	3.99 ± 4.88^b^
	Average	5.06 ± 2.59^**A**^	11.18 ± 2.33^**B**^	6.11 ± 2.72^**A**^	7.75 ± 4.07^**AB**^

III	Day 1	3.76 ± 3.53^a^	10.96 ± 5.09^b^	1.19 ± 0.92^a^	14.48 ± 3.43^b^
	Day 2	4.57 ± 3.10^a^	8.71 ± 4.06^b^	3.85 ± 5.06^a^	9.44 ± 2.62^b^
	Day 3	7.43 ± 4.93^a^	5.81 ± 4.23^b^	6.45 ± 2.91^a^	4.60 ± 1.80^b^
	Day 4	12.05 ± 5.41^a^	8.58 ± 5.64^b^	7.46 ± 5.68^a^	9.28 ± 5.80^b^
	Average	6.95 ± 3.24^**A**^	8.51 ± 1.82^**AB**^	4.73 ± 2.43^**A**^	9.45 ± 3.49^**AB**^

Values are mean ± SD, I = daily, II = once a week, and III = alternatively.

Values with similar superscripts in a column for four days of application (a or b) and for average values (A or B) at each mode of application indicate no significant difference.

Values with different superscripts in a row for each sex for four days of application (a-b) and for average values (A-B) at each mode of application indicate significant difference at *P* ≤ 0.05.

**Table 3:** Mean daily food consumption by *Rattus rattus *in response to 20% eucalyptus oil applied as spray.

Mode of application	Days of application	Mean daily food consumption (g/100 g bw)			
		Female rats (*n* = 6) (body wt = 128.33 ± 10.67 g)		Male rats (*n* = 6) (body wt = 151.66 ± 20.34 g)	
		Treatment side	Untreated side	Treatment side	Untreated side
I	Day 1	2.61 ± 4.22^a^	6.62 ± 5.01^b^	4.58 ± 4.63^a^	10.64 ± 4.01^b^
	Day 2	10.59 ± 5.31^a^	14.15 ± 1.02^b^	8.37 ± 3.71^a^	14.95 ± 2.70^b^
	Day 3	5.67 ± 3.61^a^	11.64 ± 5.70^b^	3.42 ± 3.11^a^	8.46 ± 4.12^b^
	Day 4	2.21 ± 1.45^a^	8.66 ± 5.14^b^	3.04 ± 4.33^a^	11.33 ± 2.27^b^
	Average	5.27 ± 3.35^**A**^	10.26 ± 2.86^**B**^	4.85 ± 2.10^**A**^	11.34 ± 2.33^**B**^

II	Day 1	1.18 ± 2.01^a^	13.49 ± 4.49^b^	3.80 ± 4.55^a^	10.5 ± 2.16^b^
	Day 2	3.78 ± 4.08^a^	11.32 ± 4.82^b^	4.68 ± 5.82^a^	10.51 ± 3.89^b^
	Day 3	3.90 ± 3.22^a^	6.94 ± 5.48^b^	6.15 ± 7.71^a^	6.92 ± 2.59^b^
	Day 4	7.34 ± 4.57^a^	5.74 ± 7.23^b^	5.54 ± 5.69^a^	7.00 ± 5.39^b^
	Average	4.05 ± 2.18^**A**^	9.37 ± 3.15^**B**^	5.04 ± 0.88^**A**^	8.73 ± 1.77^**AB**^

III	Day 1	2.29 ± 2.99^a^	13.24 ± 3.03^b^	4.15 ± 2.16^a^	13.62 ± 4.82^b^
	Day 2	2.11 ± 2.15^a^	10.17 ± 5.63^b^	0.8 ± 1.07^a^	8.26 ± 3.75^b^
	Day 3	5.78 ± 5.22^a^	11.58 ± 6.47^b^	4.17 ± 3.78^a^	8.31 ± 4.24^b^
	Day 4	2.81 ± 3.51^a^	9.11 ± 5.97^b^	5.84 ± 4.86^a^	4.44 ± 3.21^b^
	Average	3.24 ± 1.48^**A**^	11.02 ± 1.55^**AB**^	3.74 ± 1.83^**A**^	8.65 ± 3.26^**AB**^

Values are mean ± SD, I = daily, II = once a week, and III = alternatively.

Values with similar superscripts in a column for four days of application (a or b) and for average values (A or B) at each mode of application indicate no significant difference.

Values with different superscripts in a row for each sex for four days of application (a-b) and for average values (A-B) at each mode of application indicate significant difference at *P* ≤ 0.05.

**Table 4:** Percent repellency with eucalyptus oil applied as spray using three different concentrations against *Rattus rattus*.

Mode of application	Days of application	Percent repellency					
		5%		10%		20%	
		Female rats (*n* = 6)	Male rats (*n* = 6)	Female rats (*n* = 6)	Male rats (*n* = 6)	Female rats (*n* = 6)	Male rats (*n* = 6)
I	Day 1	84.47 ± 28.14^a^	82.98 ± 20.55^a^	64.79 ± 42.73^a^	83.13 ± 37.18^a^	62.54 ± 44.80^a^	64.39 ± 36.91^a^
	Day 2	88.02 ± 9.91^a^	75.36 ± 21.92^a^	91.46 ± 9.90^a^	76.99 ± 31.96^a^	29.10 ± 33.27^a^	43.30 ± 29.94^a^
	Day 3	76.13 ± 37.52^a^	54.38 ± 46.45^a^	63.96 ± 34.45^a^	59.36 ± 33.00^a^	54.87 ± 29.31^a^	63.68 ± 23.41^a^
	Day 4	86.12 ± 21.12^a^	88.47 ± 26.69^a^	71.08 ± 34.40^a^	72.26 ± 40.40^a^	58.12 ± 32.28^a^	74.68 ± 35.68^a^
	Average	83.68 ± 4.53^**A**^	75.29 ± 12.94^**A**^	72.82 ± 11.10^**A**^	72.93 ± 8.73^**A**^	51.15 ± 13.02^**A**^	61.51 ± 11.38^**A**^

II	Day 1	56.48 ± 42.51^a^	78.27 ± 26.69^a^	76.24 ± 36.25^a^	80.07 ± 28.35^a^	85.37 ± 25.68^a^	64.12 ± 42.80^a^
	Day 2	56.56 ± 37.17^a^	54.15 ± 38.84^a^	79.05 ± 35.89^a^	50.82 ± 36.93^ab^	60.10 ± 44.72^ab^	60.53 ± 44.73^a^
	Day 3	60.68 ± 43.23^a^	61.35 ± 41.87^a^	32.52 ± 42.07^ab^	13.15 ± 29.41^b^	43.68 ± 43.98^bc^	57.84 ± 41.90^ab^
	Day 4	47.54 ± 41.32^a^	38.58 ± 38.89^a^	36.24 ± 38.92^ab^	30.19 ± 43.04^b^	20.50 ± 29.51^c^	49.67 ± 39.28^a^
	Average	55.31 ± 4.79^**B**^	58.08 ± 14.26^**B**^	56.01 ± 21.69^**B**^	43.55 ± 24.94^**B**^	52.41 ± 23.66^**A**^	58.04 ± 5.32^**A**^

III	Day 1	42.31 ± 22.28^a^	36.58 ± 24.41^a^	59.90 ± 38.95^a^	92.34 ± 6.16^a^	77.54 ± 34.41^a^	62.32 ± 34.06^a^
	Day 2	72.80 ± 34.33^a^	60.79 ± 21.30^a^	46.12 ± 33.78^a^	70.08 ± 37.76^a^	62.98 ± 45.20^a^	85.80 ± 22.19^a^
	Day 3	57.04 ± 42.37^a^	43.07 ± 45.24^a^	19.81 ± 20.87^ab^	8.10 ± 11.46^b^	51.98 ± 45.27^a^	50.76 ± 43.24^a^
	Day 4	70.21 ± 33.99^a^	57.77 ± 30.93^a^	12.88 ± 20.61^a^	45.27 ± 42.73^a^	50.24 ± 49.75^a^	43.39 ± 44.80^a^
	Average	60.59 ± 12.12^**B**^	49.55 ± 10.05^**B**^	34.67 ± 19.12^**C**^	53.94 ± 31.27^**ABC**^	60.68 ± 10.88^**A**^	60.56 ± 16.05^**A**^

Values are mean ± SD, I = daily, II = once a week, and III = alternatively.

Values with similar superscripts in the column for four days (a or b) and for average values (A, B, or C) at each mode of application indicate no significant difference in percent repellency.

Values with different superscripts in a row for four days (a-b) and for average values (A–C) at each mode of application indicate significant difference in percent repellency between the two sexes at *P* ≤ 0.05.
